# Glycan repositioning of influenza hemagglutinin stem facilitates the elicitation of protective cross-group antibody responses

**DOI:** 10.1038/s41467-020-14579-4

**Published:** 2020-02-07

**Authors:** Seyhan Boyoglu-Barnum, Geoffrey B. Hutchinson, Jeffrey C. Boyington, Syed M. Moin, Rebecca A. Gillespie, Yaroslav Tsybovsky, Tyler Stephens, John R. Vaile, Julia Lederhofer, Kizzmekia S. Corbett, Brian E. Fisher, Hadi M. Yassine, Sarah F. Andrews, Michelle C. Crank, Adrian B. McDermott, John R. Mascola, Barney S. Graham, Masaru Kanekiyo

**Affiliations:** 1 Vaccine Research Center, National Institute of Allergy and Infectious Diseases, National Institutes of Health, 40 Convent Drive, Bethesda, MD 20892 USA; 2 Electron Microscopy Laboratory, Cancer Research Technology Program, Frederick National Laboratory for Cancer Research sponsored by the National Cancer Institute, ATRF, 8560 Progressive Drive, Frederick, MD 21702 USA; 3 Biomedical Research Center, Qatar University, New Research Complex Zone 5, Doha, Qatar

**Keywords:** Glycoproteins, Humoral immunity, Vaccines, Influenza virus

## Abstract

The conserved hemagglutinin (HA) stem has been a focus of universal influenza vaccine efforts. Influenza A group 1 HA stem-nanoparticles have been demonstrated to confer heterosubtypic protection in animals; however, the protection does not extend to group 2 viruses, due in part to differences in glycosylation between group 1 and 2 stems. Here, we show that introducing the group 2 glycan at Asn38_HA1_ to a group 1 stem-nanoparticle (gN38 variant) based on A/New Caledonia/20/99 (H1N1) broadens antibody responses to cross-react with group 2 HAs. Immunoglobulins elicited by the gN38 variant provide complete protection against group 2 H7N9 virus infection, while the variant loses protection against a group 1 H5N1 virus. The N38_HA1_ glycan thus is pivotal in directing antibody responses by controlling access to group-determining stem epitopes. Precise targeting of stem-directed antibody responses to the site of vulnerability by glycan repositioning may be a step towards achieving cross-group influenza protection.

## Abstract (web summary)

Influenza virus hemagglutinin (HA) stem between group 1 and 2 viruses has different glycosylation patterns, likely hampering cross-group protection. Here, Boyoglu-Barnum et al. show that introducing a group 2 glycan into a group 1 stem nanoparticle vaccine broadens antibody responses in mice to cross-react with group 2 HAs.

## Introduction

Influenza virus is a rapidly-evolving pathogen that continues to pose a substantial public health burden worldwide despite the availability of licensed vaccines, underscoring the need for more efficacious vaccines. Hemagglutinin (HA) is the most prevalent influenza viral surface glycoprotein and engages sialic acid moieties on host cell surface to mediate viral attachment, virus–host membrane fusion and infection^1,2^. Thus, HA is a primary target for neutralizing antibodies and vaccine design. Based on phylogenetic analysis, influenza A virus HAs can be divided into 18 subtypes (H1–H18) and classified as either group 1 or group 2 (refs. ^3,4^). Since antibodies that cross-react to multiple HA subtypes within a group and/or across groups are of great interest for universal influenza vaccine efforts^5,6^, numerous studies to elucidate the molecular and structural basis for antibody-mediated neutralization have been reported in the past few decades (reviewed in refs. ^7–9^). These studies provide the rationale for building vaccines to focus on the conserved antigenic supersites of the virus that are recurrently targeted by broadly neutralizing antibodies (bNAbs) in multiple individuals.

Current influenza vaccines provide protection primarily through the induction of neutralizing antibodies against the immunodominant globular head region of the HA which undergoes continuous antigenic drift, and hence, is highly variable among different isolates. As a result, current seasonal vaccines are effective only against antigenically-matched viruses and minimally protective against antigenically mismatched viruses or pandemic strains. In contrast, the immunologically subdominant stem region of HA is highly conserved among and across subtypes and targeted by a number of bNAbs capable of cross-reacting with multiple viral subtypes^10–22^. Thus, directing vaccine-induced antibody responses to the conserved stem region has been a major strategy for universal influenza vaccine development efforts^23–28^.

To date, several studies have shown that engineered HA stem-based immunogens that lack the immunodominant globular head domain conferred protection against heterosubtypic influenza virus infections in animal models^26,29–34^. One such vaccine that displays the structurally stabilized group 1 HA stem trimers on self-assembling nanoparticles conferred antibody-mediated heterosubtypic H5N1 protection in mice and ferrets^32^. However, the molecular properties of HA stem immunogens required to confer protection across groups has not been fully elucidated. In the last decade, a number of HA stem-directed human bNAbs have been discovered and structurally characterized^10–14,18,20–22^, providing deeper structural understanding of antibody recognition of the HA stem supersite. Although several exceptional bNAbs recognize and neutralize viruses across groups, many other bNAbs only neutralize viruses across subtypes within either group 1 or 2. Differences in glycosylation sites on the HA stem between group 1 and 2 have been suggested to have a major impact on accessibility of the stem epitope and may pose a substantial hurdle to eliciting antibodies with cross-group reactivity^22,35^. Although most group 1 HAs possess an *N*-linked glycosylation site at N33_HA1_, most group 2 HAs have a glycan at N38_HA1_ with the exception of H4 and H14 subtypes. Importantly, some of the most prevalent human HA stem-directed antibodies that broadly recognize group 1 HAs such as those derived from the V_H_1-69 lineage^12,14–16,36–38^ generally do not tolerate the group 2 glycan attached to N38_HA1_ as the location of this glycan overlaps with the epitope. While this group 2 glycan at N38_HA1_ poses a major challenge for group 1-restricted bNAbs to gain cross-reactivity to group 2 HAs, its role in eliciting cross-group antibody responses and shaping antibody specificity are not fully understood.

In this study, we either introduce the group 2-like *N*-linked glycosylation site at the position 38_HA1_ on the group 1 HA stem nanoparticle based on A/New Caledonia/20/99 (H1N1) (NC99) or replace the parental His residue at 38_HA1_ with a bulkier Arg, and then assess antigenic and immunological consequences of these changes. These variant nanoparticles are characterized for their recognition by group 1-, group 2- and cross-group stem-directed bNAbs. The specificity of antibody responses and protective immune responses elicited by the variant stem nanoparticles to homologous H1N1 NC99, heterosubtypic group 1 H5N1 and group 2 H7N9 viruses are also assessed. Collectively, these modifications to position 38_HA1_ substantially alter both the antigenicity and immunogenicity profile of group 1 stem nanoparticles. Access of group 1-specific bNAbs to the HA stem supersite is abrogated, while access of cross-group bNAbs is not impeded, which results in the elicitation of antibodies that recognize group 2 HA in immunized mice. Furthermore, immunoglobulins (Igs) purified from mice immunized with group 2 glycan variant nanoparticle provide complete protection against H7N9 infection in mice when the Igs are passively administered, suggesting that the position 38_HA1_ modification may be a pivotal step for achieving cross-group influenza protection.

## Results

### Characterization of group 1 H1 stem nanoparticle variants

**Fig. 1:**
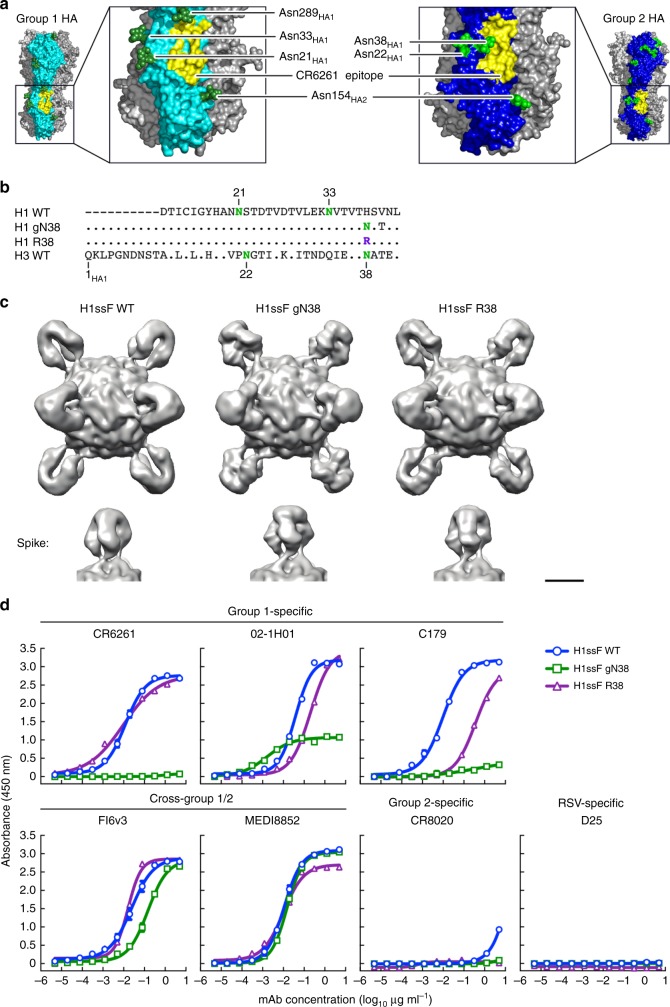
Design and characterization of group 1 H1 stem nanoparticle variants. **a** Comparison of *N*-linked glycosylation pattern of group 1 and group 2 HA stem. Surface renderings of HA trimer for a representative group 1 HA (A/Solomon Islands/06 (H1N1), PDB: 3SM5, left) and representative group 2 HA (A/Finland/486/04 (H3N2), PDB: 2YP5, right). One monomer is colored in blue for visibility. The terminal *N*-acetylglucosamine (GlcNAc) moiety for each glycan is modeled in green and residue positions are labeled according to the H3 numbering. Yellow areas designate the approximate location of the CR6261 epitope on each HA. **b** Amino acid sequence alignment of N-terminal portion of HA1 of H1 (A/New Caledonia/20/99), H3 (A/Finland/486/04) and designed variants gN38 and R38. Dots and dashes indicate residues identical to H1 and gaps, respectively. **c** Cryo-electron microscopy structures of H1 stem nanoparticle (H1ssF WT) and its variants H1ssF gN38 and H1ssF R38. Nanoparticles are depicted along the 2-fold symmetry axis. Side views of HA stem trimeric spikes are shown below each nanoparticle. The maps were low-pass filtered to a resolution of 7 Å for comparison. Scale bar indicates 5 nm. **d** Antigenicity of H1 stem nanoparticle variants. ELISA binding curves are shown for mAbs specific to group 1 stem (CR6261, 02-1H01 and C179), group 2 stem (CR8020), both group 1 and 2 stems (FI6v3, MEDI8852), or irrelevant RSV F protein (D25). Source data are provided as Source Data file.

Most influenza A HAs possess 3 or 4 potential *N*-linked glycosylation sites on the stem region of each protomer. While the locations of these glycans are generally conserved among different subtypes within each group, one of the glycosylation sites is noticeably different between group 1 and group 2 HAs (N33_HA1_ and N38_HA1_, respectively) (Fig. 1a). The group 2 glycan at N38_HA1_ is located within the canonical group 1-specific stem bNAb epitope exemplified by CR6261 (refs. ^12,15^) (Fig. 1a). To empirically test the impact of this group 2 glycan at N38_HA1_ in the context of group 1 stem, we altered a group 1 H1 stem nanoparticle (H1ssF) based on A/New Caledonia/20/1999 (NC99)^32^ with two point mutations (H38N and V40T in HA1, H3 numbering^39^) to introduce the group 2-like *N*-linked glycosylation sequon (H1ssF gN38) (Fig. 1b). In addition, we also made a variant H1ssF possessing Arg at 38_HA1_ (H1ssF R38) to test whether replacing the wild-type (WT) His with a larger side chain (H38R in HA1) alters the antigenicity and immunogenicity of the group 1 stem (Fig. 1b). The H1ssF and its gN38 and R38 variant nanoparticles were produced in mammalian cells by transient transfection and purified by anion exchange chromatography followed by size exclusion chromatography^32^, which resulted in a distinct peak consistent with ~1.2 MDa particles (Supplementary Fig. 1a). Negative-stain electron microscopy of purified nanoparticles followed by reference-free 2D classification and averaging revealed spherical particles with four to six visible regularly-spaced protruding spikes depending on the projection, consistent with the octahedral symmetry of the ferritin nanoparticle (Supplementary Fig. 1b). Cryo-electron microscopy analysis further resolved the structures of these nanoparticles to a resolution of 4.7–6.4 Å (Fig. 1c). All three nanoparticles were homogeneous and contained a well-defined 12–13 nm diameter ferritin core and clearly visible trimeric HA stem spikes with heights of ~7 nm, demonstrating that neither the gN38, nor the R38 modification affected the particle formation or trimerization of HA stem spikes (Fig. 1c). To test whether the introduction of the potential glycosylation site at 38_HA1_ resulted in glycosylation, we performed SDS-PAGE analysis of the purified H1ssF variants. While H1ssF WT and R38 resulted in approximately the same mobility shift in the reduced SDS-PAGE, H1ssF gN38 migrated higher than the other variants, indicating a larger molecular weight for this construct. This difference in mobility shift was not apparent when the proteins were treated with a deglycosydase PNGase F which removes glycans attached to Asn residues, suggesting the presence of additional *N*-linked glycans in H1ssF gN38 accounted for the observed difference in mobility shift (Supplementary Fig. 1c). To test antigenicity of the stem nanoparticle variants, we used group 1-specific V_H_1-69-derived bNAbs CR6261 (refs. ^12,15^) and 02-1H01 (ref. ^40^), murine group 1-specific antibody C179 (ref. ^41^), cross-group reactive FI6v3 (ref. ^10^) and MEDI8852 (ref. ^21^) stem-directed bNAbs, and group 2-specific CR8020 (ref. ^42^). As expected, unmodified H1ssF (WT) was recognized by group 1-specific CR6261, 02-1H01, C179 and cross-group bNAbs similarly by ELISA (Fig. 1d). Analogous to H1ssF WT, H1ssF R38 reacted with CR6261, FI6v3, and MEDI8852, but reactivity to 02-1H01 and C179 was lower than the H1ssF WT. However, while retaining reactivity to cross-group bNAbs, the reactivity of the H1ssF gN38 variant to group 1-specific bNAbs was severely compromised (Fig. 1d). None of these nanoparticles showed binding to either the group 2-specific CR8020 or the anti-RSV D25 control antibody^43^. These results confirm that introducing the group 2-like glycan at N38_HA1_ on the group 1 HA stem nanoparticle alters antigenicity of group 1 stem by selectively limiting access of strictly group 1-specific stem-directed antibodies.

### Immunogenicity of H1 stem nanoparticle variants in mice

**Fig. 2:**
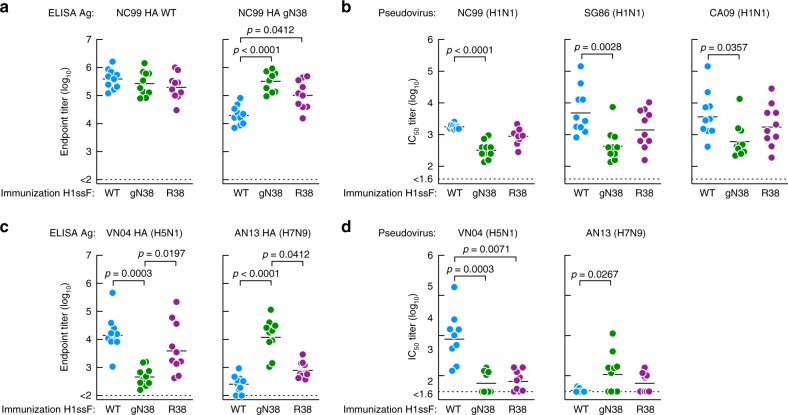
Immunogenicity of H1 stem nanoparticle variants in mice. BALB/c mice (*N* = 10) were immunized with 2 µg of H1ssF WT, H1ssF gN38 or H1ssF R38 on weeks 0, 4, and 8. Serum antibody titers to HAs were measured at 2 weeks after the third immunization (week 10). **a** ELISA endpoint titers to H1N1 NC99 HA (left) and its gN38 variant (right). **b** Serum neutralizing antibody titers to NC99, SG86, and CA09 (H1N1) pseudoviruses. Shown are half-maximal inhibitory dilution of serum (IC_50_). **c** ELISA endpoint titers to H5N1 VN04 (left) and H7N9 AN13 HAs (right). **d** Serum neutralizing antibody titers to H5N1 VN04 and H7N9 AN13 pseudoviruses. Dotted lines indicate the lower detection limit of the assay. Data are presented as scattered dot plots with horizontal lines indicating geometric mean for each group. Statistical analysis was carried out by using nonparametric Kruskal–Wallis test with Dunn’s multiple comparisons. Displayed results are representative of two independent experiments (H1ssF WT and H1ssF gN38) or based on one experiment (H1ssF R38). Source data are provided as Source Data file.

We next sought to evaluate the impact of the modifications at position 38_HA1_ on H1ssF on immunogenicity in vivo. Mice were immunized three times with H1ssF WT, H1ssF gN38 or H1ssF R38 formulated with Sigma Adjuvant System (SAS) at weeks 0, 4, and 8. All the H1ssF variants elicited robust antibody responses to the homologous A/New Caledonia/20/99 H1N1 (NC99) HA (Fig. 2a, left) since these H1ssF variants shared most of the solvent-exposed HA stem surface for immune recognition. However, when we tested serum recognition of the NC99 HA harboring the gN38 mutation (NC99 HA gN38; H38N/V40T in HA1), the antibody titers in mice immunized with H1ssF gN38 and R38 were significantly higher than that of H1ssF WT-immunized mice (*p* < 0.0001, and *p* = 0.0412, nonparametric Kruskal–Wallis test with Dunn’s multiple comparisons, respectively) (Fig. 2a, right). This indicated that both H1ssF gN38 and R38 variants directed the antibody responses that were capable of tolerating the glycan at position 38_HA1_. Interestingly, the pseudovirus neutralization half-maximal inhibitory concentration titers (IC_50_) to homotypic NC99 and H1N1 A/Singapore/6/86 (SG86) strains elicited by H1ssF WT were higher than the titers elicited by the H1ssF gN38 and R38 variants (Fig. 2b). The apparent inverse correlation between binding titers and neutralization titers suggests that the majority of neutralizing antibodies elicited by H1ssF WT were blocked by the gN38 mutation. Moreover, H1ssF gN38 elicited significantly lower antibody responses to heterosubtypic group 1 HA from H5N1 A/Vietnam/1203/04 (VN04) virus compared with that of H1ssF WT and H1ssF R38 (*p* = 0.0003 and *p* = 0.0197, nonparametric Kruskal–Wallis test with Dunn’s multiple comparisons, respectively) (Fig. 2c, left). Similar results were observed when the immune sera were tested for a different H5 HA (A/Indonesia/5/05) (Supplementary Fig. 2a). These reduced H5-binding antibody titers in mice immunized with H1ssF gN38 also resulted in a loss of neutralizing activity against H5N1 VN04 pseudovirus (Fig. 2d, left), suggesting that the gN38 mutation masked a major heterosubtypic neutralization-sensitive epitope(s). In contrast, mice immunized with H1ssF gN38 generated substantially higher antibody responses to heterosubtypic group 2 HA from H7N9 A/Anhui/1/13 (AN13) virus than mice immunized with H1ssF WT or H1ssF R38 (*p* < 0.0001 or *p* = 0.0412, nonparametric Kruskal–Wallis test with Dunn’s multiple comparisons, respectively) (Fig. 2c, right). Similarly, immune sera obtained from H1ssF gN38-immunized mice had higher reactivity to another H7 HA (A/Shanghai/1/13) (Supplementary Fig. 2a). Furthermore, considerable virus neutralization activity against H7N9 AN13 pseudovirus was also detected in immune sera obtained from mice immunized with H1ssF gN38. However, no such activity was found in sera from mice immunized with H1ssF WT (Fig. 2d, right). Immunization with H1ssF R38 resulted in an intermediate immunogenicity profile with lower H5N1 reactivity than H1ssF WT and lower H7N9 reactivity than H1ssF gN38 (Fig. 2c, d and Supplementary Fig. 2a). Therefore, H1ssF gN38 elicited antibody responses that were distinct from that induced by H1ssF WT and cross-reacted with distantly related group 2 H7N9 virus.

We also tested immune sera from mice immunized with group 2 H7 stem nanoparticle (H7ssF)^40^ as a comparator in the ELISA against H7N9 AN13 HA. The serum H7-binding titers in H7ssF-immunized mice were significantly higher than those in H1ssF gN38-immunized mice by ~2 log_10_ (Supplementary Fig. 2b). This suggests that although the glycan repositioning of H1ssF shifts the antibody responses to be more cross-reactive with group 2 HAs, the magnitude of the response is not comparable to that induced by the genuine group 2 stem immunogen. It is important to note however, that neither the glycan-unmodified group 1 nor the group 2 stem immunogen elicits cross-group neutralizing antibody responses in mice.

### Specificity of serum neutralizing activity

**Fig. 3:**
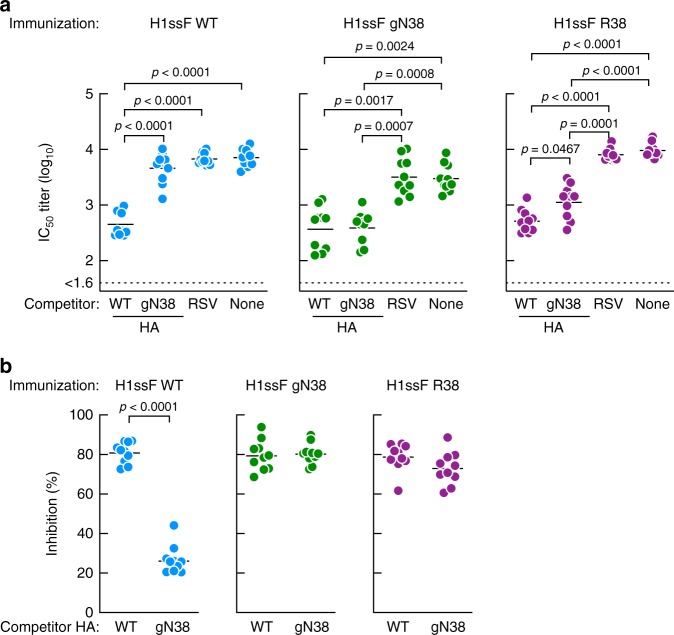
Specificity of serum neutralizing activity elicited by H1 stem nanoparticle variants. **a** Serum pseudovirus-neutralization activity in the presence of competitor proteins. Immune sera were pre-incubated with either NC99 WT HA, HA gN38, or irrelevant RSV F proteins prior to evaluation of SG86 (H1N1) pseudovirus neutralization. Serum neutralization IC_50_ titers were calculated in the absence or presence of competitor proteins. **b** Percent inhibition of virus neutralization was calculated for each competitor protein. Dotted lines indicate the lower detection limit of the assay. Data are presented as scattered dot plots with horizontal lines indicating geometric mean for each group. Statistical analysis was carried out by using nonparametric Kruskal–Wallis test with Dunn’s multiple comparisons. Displayed results are representative of two independent experiments. Source data are provided as Source Data file.

We next defined the specificity of virus neutralizing antibodies in the immune sera elicited by H1ssF WT, gN38, or R38 vaccination. To detect whether the serum neutralizing activity was targeted to the site around 38_HA1_, we performed neutralization assays in the presence of excess amounts of either NC99 HA, NC99 HA gN38, or an irrelevant RSV F protein (DS-Cav1)^44^ and assessed non-competed neutralizing activity. Expectedly, most of the neutralization activity was depleted when immune sera were pre-incubated with NC99 HA WT and only negligible reduction in neutralization was observed with DS-Cav1 in all immunization groups (Fig. 3a). Although pre-incubation with NC99 HA gN38 had a minor impact (mean ± s.d. of 26.72 ± 7.16% inhibition) on neutralization activity of immune sera obtained from mice immunized with H1ssF WT, the same treatment resulted nearly complete inhibition (80.38 ± 5.29%) in neutralization for immune sera obtained from mice immunized with H1ssF gN38 (Fig. 3a, b). Pre-incubation of immune sera obtained from mice immunized with H1ssF R38 with NC99 HA gN38 resulted in a more modest inhibition (72.97 ± 8.28%) in neutralization activity. These results indicate while H1ssF WT induces substantial neutralizing antibody titers targeting the site at or near position 38_HA1_, the majority of neutralizing antibodies elicited by H1ssF gN38 and H1ssF R38 either targets site(s) outside of position 38_HA1_ or tolerate the glycan attached to this position. Moreover, the strong competition of neutralizing activity in H1ssF g38-immunized mice by NC99 HA WT (Fig. 3a) suggests that the neutralizing epitope(s) do not require the N38 glycan for antibody recognition.

### Homotypic, heterosubtypic, and cross-group protection

**Fig. 4:**
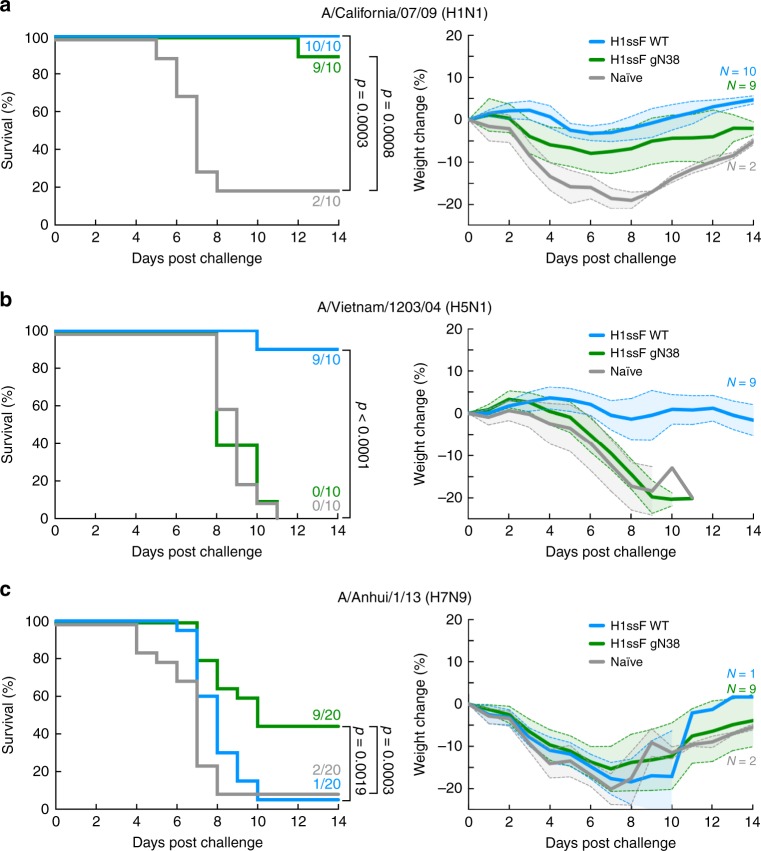
Homotypic, heterosubtypic, and cross-group protection of mice immunized with H1 stem nanoparticle variants. BALB/c mice (*N* = 10) were immunized with 2 µg of H1ssF WT, H1ssF gN38 or H1ssF R38 on weeks 0, 4, and 8, and challenged between weeks 16 and 18 with 20 × LD_50_ of A/California/07/09 (H1N1) virus (**a**), 25 × LD_50_ of A/Vietnam/1203/04 (H5N1) virus (**b**), or 10 × LD_50_ of A/Anhui/1/13 (H7N9) virus (**c**). All viruses were given intranasal inoculation. Mice were monitored twice daily for their weight and activity for 14 days post infection. Survival curve (left) and associated weight loss curve (right) were plotted for each virus challenge (**a**–**c**). For weight loss curves, lines indicate group mean ± s.d. (shaded). Statistical test to compare multiple Kaplan–Meier curves was carried out by using Mantel-Cox log-rank test with Bonferroni correction. Displayed results are representative of two independent experiments (H1N1 and H5N1) or cumulative of two experiments (H7N9). Source data are provided as Source Data file.

To assess how changes in cross-reactivity of antibody responses induced by H1ssF gN38 impact protective immunity, we carried out experimental challenge studies with multiple influenza viruses in mice. Mice were immunized three times with either H1ssF WT or H1ssF gN38 with SAS adjuvant at weeks 0, 4, and 8, and infected with either antigenically mismatched H1N1 A/California/07/09 (CA09), heterosubtypic group 1 H5N1 VN04, or heterosubtypic group 2 H7N9 AN13 virus between weeks 16 and 18. Both H1ssF WT and gN38 provided complete or near complete protection against H1N1 CA09 virus (Fig. 4a, left) with very mild weight loss compared with control animals (Fig. 4a, right). Consistent with our previous results^32^, H1ssF WT immunization conferred near complete protection against heterosubtypic group 1 H5N1 VN04 infection. However, H1ssF gN38 provided no protection against the H5N1 virus (Fig. 4b, left) and immunized mice suffered weight loss similar to that observed in naïve animals (Fig. 4b, right). This loss of heterosubtypic H5N1 protection reflected severely diminished ELISA binding titers as well as serum neutralization activity against the H5N1 virus (Fig. 2c, d). Remarkably, mice immunized with H1ssF gN38 were partially protected (45% survival) from heterosubtypic group 2 H7N9 AN13 virus infection in contrast to only 5–10% survival with either H1ssF WT or mock immunized animals (Fig. 4c). Although the survivors in the H1ssF gN38-immunized mice appeared to lose substantial weight (−13.67 ± 3.65% at the peak) upon H7N9 infection and the protection was not optimal, this improved cross-group protection provided by the H1ssF gN38 was statistically significant when compared with mice immunized with H1ssF WT or naïve control (*p* = 0.019 or *p* = 0.0003, Mantel-Cox log-rank test with Bonferroni correction, respectively) (Fig. 4c). These results suggest that even a modest increase in serum antibody cross-reactivity to group 2 heterosubtypic H7N9 virus induced by H1ssF gN38 can result in improved cross-group protective efficacy against the group 2 H7N9 virus in immunized mice.

### Cross-group protection by hyperimmune immunoglobulins

**Fig. 5:**
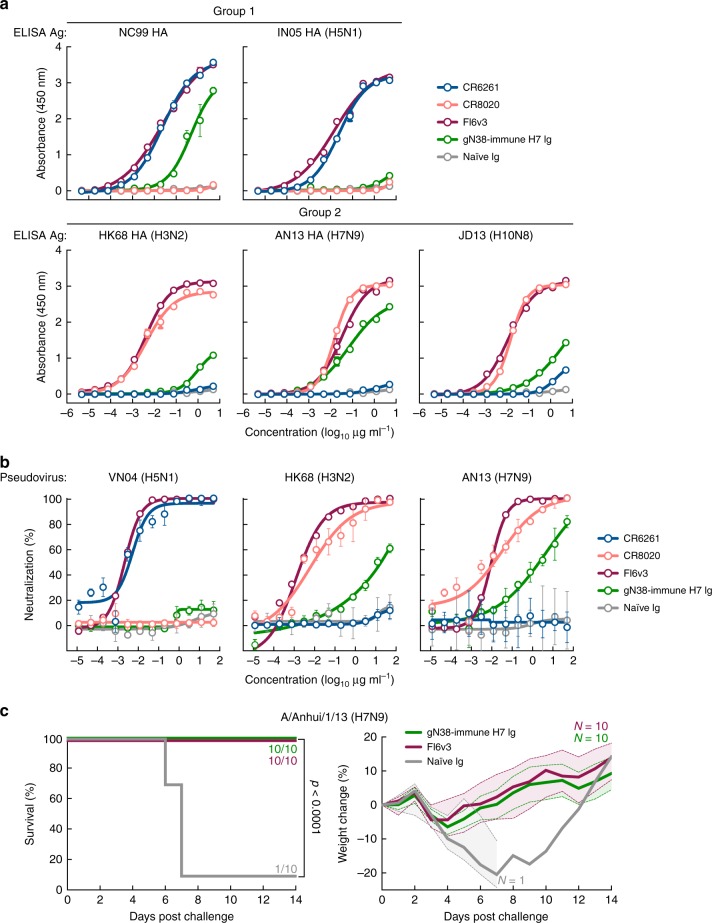
Cross-group neutralization and protective capacity of immunoglobulins isolated from mice immunized with H1 stem nanoparticle gN38 variant. Immune Ig were obtained from H1ssF gN38-immunized mice by affinity-purifying with H7 (AN13) HA. **a** ELISA binding profile of purified Ig to HA from NC99, H5N1 IN05, H3N2 HK68, H7N9 AN13, and H10N8 JD13. **b** Neutralization profile of purified Ig to H5N1 VN04 (left), H3N2 HK68 (center), and H7N9 AN13 (right) pseudoviruses. Data are shown as mean ± s.d. **c** Passive immunization of purified Ig in naïve BALB/c mice (*N* = 10). Mice were given 0.2 mg of purified Ig from either naïve or H1ssF gN38-immunized mice, or bNAb FI6v3 (0.1 mg) intraperitoneally 24 h prior to H7N9 virus challenge (10 × LD_50_). Mice were monitored twice daily for their weight and activity for 14 days post infection. Statistical test to compare multiple Kaplan–Meier curves was carried out by using Mantel-Cox log-rank test with Bonferroni correction. Displayed results are from one experiment. Source data are provided as Source Data file.

To further characterize the cross-group reactivity of serum antibodies elicited by H1ssF gN38 immunization, we affinity-purified Igs from immune sera by using recombinant H7N9 AN13 HA. The purified H7N9 AN13 HA-reactive Igs not only bound H7N9 AN13 HA but also HAs derived from other group 2 subtypes H10N8 A/Jiangxi-Donghu/346/13 (JD13) and H3N2 A/Hong Kong/1/68 (HK68) viruses, albeit with weaker affinity than to H7N9 AN13 HA (Fig. 5a). Importantly, the H7N9 AN13 HA-reactive Igs also recognized autologous NC99 HA as they were elicited by H1ssF gN38 immunization, though reactivity to H5N1 IN05 HA was negligible (Fig. 5a). Neutralizing activity of the affinity-purified Igs for H7N9 AN13 and H3N2 HK68 pseudoviruses reached an IC_50_ concentrations of 2.12 and 18.08 µg ml^−1^, respectively, but neutralizing activity against H5N1 VN04 pseudovirus was undetectable (>50 µg ml^−1^) (Fig. 5b). To assess whether the H7N9 AN13 HA-reactive Igs provide protection when prophylactically administered into naïve animals, we passively immunized mice with 0.2 mg of Igs (~10 mg kg^−1^) 24 h prior to infection with H7N9 AN13 virus. Following the virus infection, mice that received H7N9 AN13 HA-reactive Igs or control influenza bNAb FI6v3 (~5 mg kg^−1^) did not suffer substantial weight loss and survived 100% throughout the study period (Fig. 5c). In contrast, 9 out of 10 mice that received control naïve Igs lost substantial weight and had to be euthanized by 7 days post infection (Fig. 5c). Collectively these results indicate that H1ssF gN38 is capable of eliciting antibodies that cross-react with multiple group 2 subtype HAs and when these group 2 HA-reactive antibodies are given prophylactically to naïve animals they confer complete cross-group protection against otherwise lethal H7N9 virus infection.

## Discussion

Despite immense efforts to curtail influenza infections over the past several decades and the availability of licensed commercial vaccines, seasonal influenza remains a major public health burden worldwide. Furthermore, there are many other exotic virus subtypes in animal reservoirs that pose potential human pandemic threats. Sporadic outbreaks of animal influenza viruses such as H5N1, and H7N9 further accentuate the need for developing broadly protective countermeasures that are effective beyond seasonal influenza viruses to protect the public from a potentially serious pandemic. Universal influenza vaccines that elicit protective immunity across all potential subtypes and lineages of viruses would be an ideal solution to achieve this goal. The discovery in 1993 of the murine antibody C179 that targets the conserved HA stem and neutralizes multiple subtypes within group 1 influenza A viruses^41^ and the subsequent discovery of multiple human bNAbs targeting the same stem region^10–15,20,45–48^ have revealed the vulnerabilities of the HA stem supersite, making it a prime target for universal influenza vaccines^5,22–25,49,50^. Although HA stem-directed bNAbs can serve as a basis for universal influenza immunity, many published bNAbs are specific to or preferentially recognize HAs within either group 1 or group 2. Only a handful of bNAbs are capable of crossing the group boundary, suggesting a substantial bottleneck to eliciting cross-group antibody reactivity even in individuals with repeated influenza exposures. One major obstacle to overcoming the group boundary is the difference in glycosylations between group 1 and group 2 stems which differentially regulate accessibility of antibodies to the underlying epitopes^22,35^. Most of group 1 HAs possess an *N*-linked glycan at N33_HA1_, whereas this glycosylation site is shifted to N38_HA1_ in most group 2 HAs. These group-specific glycans appear to serve as gatekeepers by allowing or disallowing stem-directed B cells (and eventually antibodies) to approach, and hence shaping the antibody responses specifically toward one group over the other. Indeed, the introduction of the group 2-like glycosylation at N38_HA1_ in an H5 HA was observed to diminish binding of a canonical group 1-specific bNAb CR6261 by ~70%^15^. We have shown that introduction of this glycan blocks binding of group 1-specific antibodies but not cross-group antibodies. Substituting the position 38_HA1_ residue with Arg results in only a marginal reduction in recognition of group 1-specific antibodies, indicating that the bulky glycan moieties are needed to more fully block the access of these antibodies. Importantly, the mutual exclusivity or strong group favoritism of the HA stem-directed antibodies applies also to vaccines as neither group 1- nor group 2-based HA stem immunogens convincingly elicit protective antibody responses that overcome the group boundary in animal models^32–34,40^. Similarly, a natural Ile to Phe variation observed in human H2 HA at the position 45_HA2_ which resides in close proximity to 38_HA1_ makes it more resistant to many HA stem-specific monoclonal antibodies (mAbs) than other group 1 subtype HAs^22,49^. In the present study, we have demonstrated that by simply changing the glycosylation pattern on the stem can alter the group-specificity of vaccine-elicited antibody responses in mice. Although the gN38 variant induced cross-group protective antibody responses, the glycan modification also resulted in the complete loss of group 1 heterosubtypic protection from H5N1. We speculated that the majority of H5-reactive serum antibody in H1ssF WT-immunized mice was targeted by a site immediately adjacent to the position 38_HA1_. Although the modifications to position 38_HA1_ limited access of B cells targeting this adjacent site, the degree of inhibition appeared size dependent, with the larger gN38 modification creating more steric occlusion than the R38 modification. While the gN38/R38 modifications inhibit the access of H1/H5 cross-reactive B cells, the obstruction redirects antibody responses to a slightly shifted site which appears to be conserved between H1 and group 2 HAs, but not H5. To optimally achieve both cross-group and intra-group heterosubtypic protection, further studies are needed to fine tune the stringency of the glycan-mediated blockage on the group 1 HA stem-based immunogen. For example, partially trimming the glycans to reduce the bulkiness of the gN38, altering the position of the N38 glycan, assembling heterotypic HA stem variants like wild-type and gN38 on the same nanoparticle, or engrafting the group 1 stem residues onto the group 2 stem backbone with its natural glycan at N38_HA1_, may further broaden the antibody immune response.

In humans, the group 1 stem-directed antibody responses are largely dominated by a single V_H_ gene family, V_H_1-69 (refs. ^14,15,22,38,51–53^), both in the context of natural exposure to pandemic H1N1 infection in 2009–2010 (refs. ^36,51^) as well as vaccination with a non-circulating group 1 HA^22^. The unique hydrophobic properties of the CDR H2 encoded in most V_H_1-69 alleles has an inherent affinity to the Trp21_HA2_ pocket on the HA stem surrounded by residues including 38_HA1_ and 45_HA2_ which results in this heavy chain being overrepresented in antibodies elicited to this site^36,51^. However, this particular hydrophobic CDR H2 motif (Ile53 and Phe54) is not present in any known V_H_ genes from other species, including mice. Interestingly, the first stem-directed murine antibody C179 (ref. ^41^) interacts with the Trp21 pocket on the stem in a remarkably similar way as V_H_1-69 antibodies but by utilizing its CDR H3 loop^54^. This suggests that mice can also generate these kinds of antibodies although it is not as reproducible as the human germline-encoded V_H_1-69 response. While several murine and other animal species have been engineered to carry partial or entire human immunoglobulin loci to produce humanized antibodies, recapitulating the V_H_ gene-dependent immune responses in these animal models is still challenging^55–59^. Moreover, cross-group bNAbs often have human-specific immunogenetic constraints that could be even more difficult to recapitulate in animal models^22,35,49,60^. Although these and other caveats such as differences in the clonal deletion process, V gene usage in naïve repertoire or VDJ recombination need to be considered, in principle these ‘humanized’ animals could still be very helpful models to test V_H_-restricted responses.

Another important aspect of human influenza immunology to consider is the preexisting immunity established through either natural exposures and/or vaccinations. The complexity and individualized history of influenza exposure is impossible to replicate in current animal models. It is important to note that the vast majority of preclinical studies are performed in antigen-naïve animals. Immunological imprinting from early-life exposures has also been implicated to have a substantial impact on skewing subsequent immune response to influenza virus potentially for life^61,62^. Therefore, for influenza-naïve individuals, the order of initial antigen exposures may be more important than antigen-experienced individuals to establish influenza virus-specific B cell repertoire with desired specificity. Current vaccines deliver multiple influenza strains in a single formulation with the intent of maximizing the breadth of HA-directed protective antibody responses in adults with preexisting immunity. This may not be ideal for naïve individuals in whom the goal is to establish specific broadly cross-reactive lineages. Such lineage-targeting may be better accomplished by a narrowed set of antigens. For antigen-experienced adults, the exotic non-circulating group 2 immunogen (i.e., H7N9 split vaccine) could preferentially boost antibody responses with greater cross-group reactivity potential by avoiding dominant group 1-specific V_H_1-69 lineage antibodies^37,38,49^. Our findings in the present study suggest that the gN38 variant of the group 1 stem nanoparticle would not only mitigate the elicitation of group 1-specific antibodies, but also completely avoid boosting of head-directed, strain-specific antibody responses. This group 2-like immunogen complements our earlier development of group 1 HA stem nanoparticle, which confers intra-group heterosubtypic protection in mice and ferrets^32^, and group 2 HA stem nanoparticles, which confer homotypic protection in mice^40^. These stem immunogens could potentially be used in a heterologous prime-boost regimen to focus antibody responses to the cross-group HA stem supersite. They could also be given together in a manner analogous to the current multivalent inactivated influenza vaccines (IIV) or in combination with IIV to mitigate the inevitable risk of discordance between vaccine and circulating strains.

Together, our study shows the importance of a group-determining stem glycan on dictating the specificity of vaccine-elicited stem-directed antibody responses and offers insights on design options for more universal influenza vaccines.

## Methods

### Expression and purification of immunogens and antibodies

All HA nanoparticles were expressed in Expi293 cells (ThermoFisher Scientific) and purified by ion exchange chromatography using a HiTrap Q HP column (GE Healthcare). Briefly, cleared transfection supernatants were diluted three times with Tris-HCl (pH 8.0) buffer at a final concentration of 50 mM Tris-HCl, ~50 mM NaCl. Diluted supernatants were directly loaded on an anion exchange column (HiTrap Q HP) and captured nanoparticles were eluted with a linear gradient NaCl (50–300 mM) in 50 mM Tris-HCl (pH 8.0) buffer. Fractions containing H1ssF (H1ssF) based on A/New Caledonia/20/99 (NC99) nanoparticles were concentrated and further purified by size exclusion chromatography with a Superose 6 pg XK 16/70 column (GE Healthcare) and the purity of the materials were assessed by SDS-PAGE and blue staining (SimplyBlue SafeStain, ThermoFisher Scientific). HA trimers, DS-Cav1 trimers, and FI6v3, CR8020, CR6261, C179, 02-1H01 and MEDI8852 mAbs were also expressed in Expi293 cells and purified by Ni-NTA (HA) or Ni-NTA and Strep-Tactin (DS-Cav1) followed by size exclusion chromatography, or by protein A (mAbs). All proteins and mAbs were tested for antigenicity and specificity prior to use.

### Negative-stain electron microscopy

H1ssF nanoparticle samples were diluted to ~20 µg ml^−1^ with a buffer containing 10 mM HEPES (pH 7.0) and 150 mM NaCl. Nanoparticles were adsorbed to a freshly glow-discharged carbon-film grid for 15 s, washed with the above buffer, and stained with 0.75% uranyl formate. Micrographs were collected using the EPU software on a ThermoFisher Scientific Talos F200C electron microscope operated at 200 kV using a 4k × 4k Ceta CCD camera (pixel size of 2.5 Å). Particles were selected from the micrographs automatically using in-house written software (Y.T., unpublished) and subjected to reference-free 2D classification in Relion^63^.

### Cryogenic electron microscopy and structure determination

H1ssF nanoparticles at a concentration of ~1 mg ml^−1^ were vitrified at room temperature and 90% humidity using Vitrobot Mark IV (FEI) by applying a 2.7 µl drop to a glow-discharged holey carbon grid (Quantifoil R 1.2/1.3, gold support), blotting for 2 s, and plunging into liquid ethane cooled by liquid nitrogen. Datasets were collected using a Talos F200C electron microscope (ThermoFisher Scientific) operated at 200 kV and equipped with a side-entry cryo-holder (Gatan 626). Movies containing 40 frames each were recorded using a Falcon 3EC direct electron detector in the integrating mode at a nominal magnification of 92,000× corresponding to a pixel size of 1.58 Å. The frame exposure time was 50 ms (total movie exposure time: 2 s), and the total dose was 60 e/A^2^. The defocus range was 1.5–4 µm underfocus. A total of 118, 248, and 106 movies were recorded for the H1ssF WT, H1ssF gN38, and H1ssF R38, respectively. Motion correction was performed using MotionCor2 (ref. ^64^). Ctffind 4.1 was used to estimate the defocus of motion-corrected micrographs^65^. The resulting micrographs and the ctffind output were examined, and low-quality images were excluded from further processing. Approximately 2000 particles were picked manually for each nanoparticle from these micrographs and subjected to 2D classification in Relion 2.1 (ref. ^63^). Selected resulting classes were low-pass filtered and used as templates for automatic particle selection as implemented in Relion. Particles were extracted into 180 × 180-pixel (H1ssF WT, H1ssF gN38) or 200 × 200-pixel (H1ssF R38) boxes. The resulting particle stacks contained 66,602, 168,964, and 68,863 particles, respectively, for H1ssF WT, H1ssF gN38, and H1ssF R38. The particles were subjected to two rounds of 2D classification with selection of best-looking classes. Initial three-dimensional models were obtained from these classes in EMAN2 (ref. ^66^). Model refinement was performed in Relion 2.1 with octahedral symmetry imposed, which was followed by three-dimensional classification. The best-looking 3D class was selected, and the refinement was repeated. A total of 51,586, 33,531, and 13,539 particles contributed to the final cryo-EM map of H1ssF WT, H1ssF gN38, and H1ssF R38, respectively. The 3D maps were sharpened by applying a negative B-factor (−500 Å^2^ for H1ssF WT, −300 Å^2^ for H1ssF gN38, −200 Å^2^ for H1ssF R38) during post processing. The resolutions determined according to the gold-standard approach using a Fourier shell correlation threshold of 0.143 were 6.3 Å (H1ssF WT), 4.7 Å (H1ssF gN38), and 6.4 Å (H1ssF R38). Local resolution was estimated in Relion. The strongest EM density, which corresponded to the highest resolution, was observed in the ferritin core. HA stem spikes were observed at lower map thresholds, and the resolution in these areas was lower due to higher flexibility. Figures were prepared using UCSF Chimera^67^.

### ELISA

ELISA was used to measure binding of H1ssF WT, H1ssF gN38, and H1ssF R38 nanoparticles to mAbs CR6261, CR8020, MEDI8552, FI6v3, C179, 02-1H01, and D25 (anti-RSV). 96-well plates were coated with 2 μg ml^−1^ H1ssF WT, H1ssF gN38, and H1ssF R38 (0.1 ml per well) and incubated at 4 °C overnight. For testing HA-specific IgG levels in immune sera, plates were coated with 2 μg ml^−1^ of recombinant HA proteins derived from H1 NC99 WT, H1 NC99 gN38, H5 IN05, and H7 SH13. Plates were then blocked with PBS containing 5% skim milk at 37 °C for 1 h. Monoclonal antibodies and immune sera were serially diluted in four-fold steps and added to the wells for an hour. Horseradish peroxidase (HRP)-conjugated anti-human or anti-mouse IgG (Southern Biotech) was added and incubated at 37 °C for 1 h. The wells were developed with 3,3′,5′,5-tetramethylbenzidine (TMB) substrate (KPL), and the reactions were stopped by adding 1 M H_2_SO_4_ before measuring absorbance at 450 nm with a Spectramax Paradigm plate reader (Molecular Devices).

### Animal experiments

All animal experiments were reviewed and approved by the Institutional Animal Care and Use Committee of the VRC, NIAID, NIH. All animals were housed and cared for in accordance with local, state, federal, and institutional policies of NIH and American Association for Accreditation of Laboratory Animal Care.

### Immunization and challenge studies

BALB/cJ mice (Jackson Laboratory) were immunized intramuscularly (IM) with 2 μg of H1ssF WT, H1ssF gN38 and H1ssF R38 with Sigma Adjuvant System (SAS) on weeks 0, 4, and 8 (*N* = 10). Mice were given 50 μl into each hind leg. Serum samples were collected before and after each immunization and tested for the immunogenicity by ELISA and pseudovirus-neutralization assays. For the challenge studies, mice infected with 10–20 times 50% lethal dose (LD_50_) of H1N1 A/California/07/09, H5N1 A/Vietnam/1203/04 or H7N9 A/Anhui/1/13 virus intranasally at Bioqual. The animals were monitored twice daily for development of clinical signs and weighed daily for 14 days. Any animals that had lost 20% or more of their initial body weight were euthanized.

### Pseudovirus-neutralization assays

Pseudovirus-neutralization assays were carried out using luciferase encoding lentiviruses pseudotyped with influenza HA and NA^68,69^. HA and NA sequences used to generate pseudoviruses were derived from A/New Caledonia/20/99 (H1N1), A/Singapore/6/86 (H1N1), A/California/4/09 (H1N1), A/Vietnam/1203/04 (H5N1), A/Anhui/1/13 (H7N9), and A/Hong Kong/1/68 (H3N2). 293T cells were cotransfected with pCMV-ΔR8.2 (lentiviral backbone) and pHR’-CMV-Luc (reporter genome) plasmids along with plasmids encoding desired HA and corresponding NA, and human transmembrane serine protease 2 (TMPRSS2) by the calcium-phosphate method (Promega). After overnight incubation, wells were washed, and replenished with fresh medium. Forty eight hours later, supernatants were harvested, filtered through a 0.45 µm, aliquoted, and frozen at −80 °C until use. Each pseudovirus stock was titrated prior to use in neutralization assays. Mouse sera were treated with receptor destroying enzyme (RDE (II); SEIKEN Accurate Chemical and Scientific) and heat-inactivated before subjecting to the assays. Immune sera or monoclonal antibodies were serially diluted and incubated with pre-titrated HA-NA pseudotyped viruses for 30 min at room temperature. Serum-pseudovirus mixture was then transferred to 96-well white/black isoplates (PerkinElmer), and 12,000 293 A cells were added into each sample well of the plate. After overnight incubation at 37 °C, wells were supplemented with 100 μl of fresh Dulbecco’s modified Eagle medium including 5% fetal bovine serum (Fisher Scientific), and 5000 units ml^−1^ penicillin-streptomycin (Gibco), and the plates were incubated in a static 37 °C, 5% CO_2_, humidified incubator for 48 h. Cells were lysed with cell culture lysis buffer (Promega) and luciferase activity in the lysate was measured using Luciferase kit (Promega). Luminescence was measured with a Spectramax L luminometer (Molecular Devices). For neutralization competition assays, mouse immune sera were pre-incubated with NC99 WT HA, gN38 HA, or control RSV F protein (at a final concentration of 50 µg ml^−1^) at RT for 1 h prior to perform the pseudovirus-neutralization assays described above. Serum dilution or antibody concentration that gives 50% neutralization (IC_50_) values were calculated from neutralization curves (four-parameter nonlinear regression model) and plotted with GraphPad Prism 8.

### Purification of polyclonal Ig

To generate hyperimmune Ig, 100 female BALB/cJ mice (6–8 weeks old; Jackson Laboratory) were immunized three times with H1ssF gN38 protein (2 μg per immunization with SAS) at weeks 0, 4, and 8 and sera were collected at 1 and 2 weeks after the last immunization prior to terminal bleed at 3 weeks after the last immunization. Briefly, 10 mg H7 AN13 HA protein was immobilized on NHS-Activated Agarose Dry Resin (Thermo Scientific) for 2 h at RT. After coupling, the resin was washed three times with PBS, then pooled immune sera was added. The column was incubated overnight at 4 °C. After washing the column briefly, captured antibodies were eluted with low-pH IgG elution buffer (Thermo Scientific) and the eluates containing H7-specific antibodies were immediately neutralized by adding 1 M Tris-HCl (pH 8.0) at a final concentration of 100 mM. Purified H7-specific antibodies were dialyzed two times against PBS, concentrated to ~1.0 mg ml^−1^ and stored in −80 °C until use.

### Passive transfer studies

BALB/cAnNHsd mice (Envigo) were given a 0.1 mg of FI6v3 (~5 mg kg^−1^) or 0.2 mg of purified H7-specific or control naïve Igs (~10 mg kg^−1^) intraperitoneally (*N* = 10). Twenty-four hours later, the mice were infected intranasally with 10 × LD_50_ of H7N9 A/Anhui/01/2013 (H7N9) at Bioqual. The animals were monitored twice daily for development of clinical signs and weighed daily for 14 days. Any animals that had lost 20% or more of their initial body weight were euthanized.

### Statistical significance

All statistical analysis was performed using Prism 7 or 8 (GraphPad software). Sample sizes of animals and specific tests to determine statistical significance used are indicated in the methods and corresponding figure legends. *P* values < 0.05 were considered statistically significant.

### Reporting summary

Further information on research design is available in the Nature Research Reporting Summary linked to this article.

## Supplementary information

Supplementary Information

Reporting Summary

## Data Availability

All data generated or analyzed during this study are included in this published article and available in a Source Data file. The source data underlying Figs. 1d, 2a–d, 3a, b, 4a–c, and 5a–c, and Supplementary Figs. 1a, and 2a, b, and uncropped gel image of Supplementary Fig. 1c are provided as a Source Data file. The H1ssF constructs used in this study have been deposited in the NCBI GenBank under accession numbers MN585111–MN585113. The Cryo-EM maps described in this study have been deposited in the EM Data Bank (EMDB) under accession numbers EMD-20911–EMD-20913.

## Source data

Source Data
