# Supplementary material for: Glycan repositioning of influenza hemagglutinin stem facilitates the elicitation of protective cross-group antibody responses
Source: Nat Commun. 2020 Feb 7;11:791. doi: 10.1038/s41467-020-14579-4 (PMC7005838; doi:10.1038/s41467-020-14579-4)
Supplement: Supplementary file 1 — Supplementary Information [file 41467_2020_14579_MOESM1_ESM.pdf]

Supplementary Information

Glycan repositioning of influenza hemagglutinin stem facilitates the elicitation of protective cross-group antibody responses

Boyoglu-Barnum et al.

Supplementary Figures

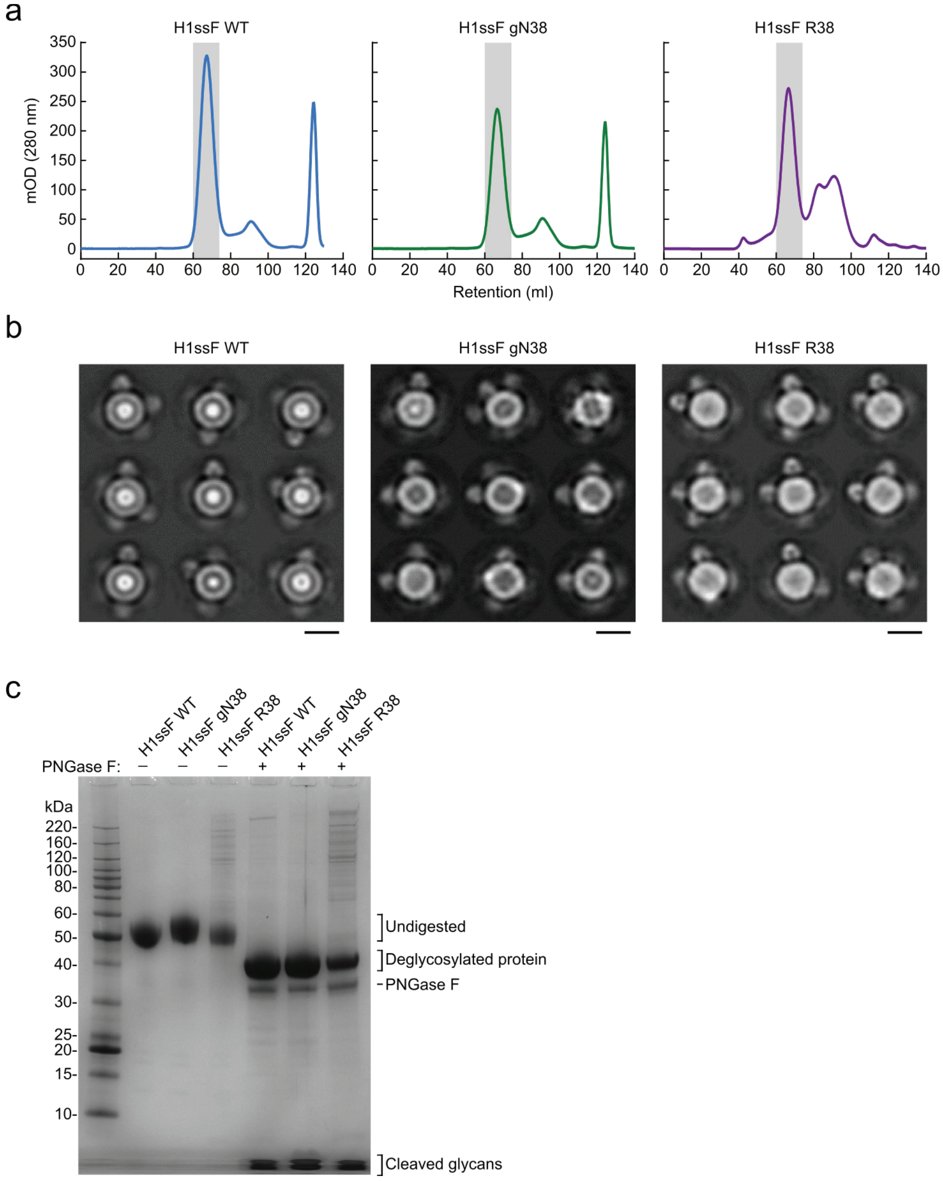

Supplementary Figure 1 | Characterization of H1 stem nanoparticles.

a, Size exclusion chromatograms of H1ssF WT, H1ssF gN38 and H1ssF R38. Size exclusion chromatography was performed using a Superose 6 pg XK 16/70 column in PBS. Shaded peaks contain assembled nanoparticles. **b**, Negative stain electron microscopy analysis of H1ssF. Representative 2D class averages are shown. Scale bar denotes 10 nm. **c**, SDS-PAGE analysis of purified H1ssF nanoparticle variants. H1ssF variants were denatured and treated with PNGase F at 37ºC for 1 hour or left untreated prior to subject SDS-PAGE. Shown is a gel image of H1ssF variants with or without PNGase F treatment.

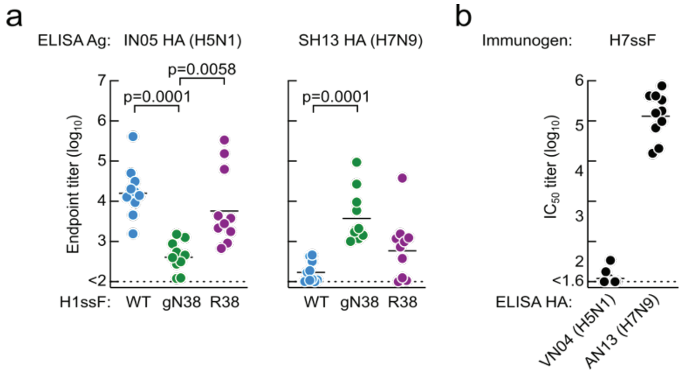

Supplementary Figure 2 | ELISA antibody titers upon immunization with H1 stem nanoparticle variants or group 2 H7 stem nanoparticle.
a, ELISA antibody titers to H5N1 IN05 (left) and H7N9 SH13 (right) HAs in mice immunized with H1ssF variants. **b**, ELISA antibody titers to H5N1 VN04 and H7N9 AN13 HAs in mice immunized with H7ssF. Data are presented as scattered dot plots with horizontal lines indicating geometric mean for each group. Statistical analysis was carried out by using nonparametric Kruskal-Wallis test with Dunn's multiple comparisons.
