# Supplementary material for: Glycan repositioning of influenza hemagglutinin stem facilitates the elicitation of protective cross-group antibody responses
Source: Nat Commun. 2020 Feb 7;11:791. doi: 10.1038/s41467-020-14579-4 (PMC7005838; doi:10.1038/s41467-020-14579-4)
Supplement: Supplementary file 2 — Reporting Summary [file 41467_2020_14579_MOESM2_ESM.pdf]

nature research

Corresponding author(s): Barney Graham & Masaru Kanekiyo

Last updated by author(s): Jan 10, 2020

Reporting Summary

Nature Research wishes to improve the reproducibility of the work that we publish. This form provides structure for consistency and transparency in reporting. For further information on Nature Research policies, see Authors & Referees and the Editorial Policy Checklist .

Statistics

For all statistical analyses, confirm that the following items are present in the figure legend, table legend, main text, or Methods section.

n/a	Confirmed	
☐	☒	The exact sample size (n) for each experimental group/condition, given as a discrete number and unit of measurement
☐	☒	A statement on whether measurements were taken from distinct samples or whether the same sample was measured repeatedly
☐	☒	The statistical test(s) used AND whether they are one- or two-sided<br>*Only common tests should be described solely by name; describe more complex techniques in the Methods section.*
☒	☐	A description of all covariates tested
☒	☐	A description of any assumptions or corrections, such as tests of normality and adjustment for multiple comparisons
☐	☒	A full description of the statistical parameters including central tendency (e.g. means) or other basic estimates (e.g. regression coefficient) AND variation (e.g. standard deviation) or associated estimates of uncertainty (e.g. confidence intervals)
☐	☒	For null hypothesis testing, the test statistic (e.g. F, t, r) with confidence intervals, effect sizes, degrees of freedom and P value noted<br>*Give P values as exact values whenever suitable.*
☒	☐	For Bayesian analysis, information on the choice of priors and Markov chain Monte Carlo settings
☒	☐	For hierarchical and complex designs, identification of the appropriate level for tests and full reporting of outcomes
☒	☐	Estimates of effect sizes (e.g. Cohen's d, Pearson's r), indicating how they were calculated

Our web collection on statistics for biologists contains articles on many of the points above.

Software and code

Policy information about availability of computer code

Data collection	*Provide a description of all commercial, open source and custom code used to collect the data in this study, specifying the version used OR state that no software was used.*
Data analysis	All data with the following exceptions were analyzed with GraphPad Prism 8.2.0<br>Cryo-EM: MotionCor2, CTFFIND4.1, RELION, RELION2.1, EMAN2<br>EM image analysis: RELION, EPU<br>Structure rendering: UCSF Chimera, Pymol

For manuscripts utilizing custom algorithms or software that are central to the research but not yet described in published literature, software must be made available to editors/reviewers. We strongly encourage code deposition in a community repository (e.g. GitHub). See the Nature Research guidelines for submitting code & software for further information.

Data

Policy information about availability of data

All manuscripts must include a data availability statement. This statement should provide the following information, where applicable:

- Accession codes, unique identifiers, or web links for publicly available datasets
- A list of figures that have associated raw data
- A description of any restrictions on data availability

All data needed to evaluate the conclusions in the paper are present in the Source Data file and/or the Supplementary Information. Cryo-EM density maps have been deposited to the EM Data Bank (EMDB) under EMD-20911–EMD-20913. Sequences are deposited to the GenBank under MN585111–MN585113. Requests for materials should be addressed to B.S.G. (bgraham@nih.gov) and M.K. (kanekiyom@nih.gov).

Field-specific reporting

Please select the one below that is the best fit for your research. If you are not sure, read the appropriate sections before making your selection.

[x] Life sciences [ ] Behavioural & social sciences [ ] Ecological, evolutionary & environmental sciences

For a reference copy of the document with all sections, see nature.com/documents/nr-reporting-summary-flat.pdf

Life sciences study design

All studies must disclose on these points even when the disclosure is negative.

Sample size	The number of animals selected for each study was chosen based on our prior experience with similar vaccine regimens and virus challenge studies. A group size of 10 animals is used. Assuming variance in immune response and/or lethality is proportional to mean for a given group (constant CV of 30%, typical for this type of experiments), a group size of 10 will give 89% power to detect 2-fold differences or 49% power to detect 1.5-fold differences between vaccine groups (set for 4) in the magnitude of the immunological parameters based on a two-tailed test of means with alpha set to 0.05 (calculation was performed by 1-way ANOVA pairwise tools at powerandsamplesize.com). A consistent 1.5- to 2-fold difference in immunological parameters is the minimum amount of difference between vaccine groups that would be relevant for biological significance.
Data exclusions	No data have been excluded.
Replication	All analyses for antibody binding, specificity, and virus neutralization assays have been performed twice or more with similar results. Most of the experiments including animal studies have been repeated with similar results. All of the data in which we could perform statistical analysis showed that the differences observed were significant and highly consistent across experiments.
Randomization	All animals were age and gender matched and randomly assigned to experimental groups.
Blinding	In vivo challenge studies were done in a blinded manner. All of the serological assays including virus neutralization assays, and structural, biochemical characterization of the particles were not performed in a blinded manner.

Reporting for specific materials, systems and methods

We require information from authors about some types of materials, experimental systems and methods used in many studies. Here, indicate whether each material, system or method listed is relevant to your study. If you are not sure if a list item applies to your research, read the appropriate section before selecting a response.

Materials & experimental systems

n/a	Involved in the study	
[ ]	[x]	Antibodies
[ ]	[x]	Eukaryotic cell lines
[x]	[ ]	Palaeontology
[ ]	[x]	Animals and other organisms
[x]	[ ]	Human research participants
[x]	[ ]	Clinical data

Methods

n/a	Involved in the study	
[x]	[ ]	ChIP-seq
[x]	[ ]	Flow cytometry
[x]	[ ]	MRI-based neuroimaging

Antibodies

Antibodies used	All the antibodies used were made recombinantly by cloning antibody heavy and light chains into the mammalian expression vectors. Antibodies were produced in mammalian cells (Expi293 cells) by transient transfection of expression vectors and purified by protein A affinity chromatography. Sequences, specificity and function of the antibodies were verified for each antibody. Secondary antibodies used in ELISA were purchased from Southern Biotech.
Validation	All the antibodies were tested for their reactivity and specificity by ELISA, BLI or other assays prior to be used in the study.

Eukaryotic cell lines

Policy information about cell lines

Cell line source(s)	Expi293 cells (Gibco)
Authentication	No authentication was performed.

Mycoplasma contamination	All cell lines employed tested negative for mycoplasma contamination in our laboratory using the MycoAlert® Mycoplasma Detection Kit (Lonza).
Commonly misidentified lines (See ICLAC register)	All cells used in the study were directly purchased from commercial vendors and not extensively passaged. No commonly misidentified cell lines were used in the study.

Animals and other organisms

Policy information about studies involving animals; ARRIVE guidelines recommended for reporting animal research

Laboratory animals	Female 4-6 week old BALB/cJ mice (Jackson Laboratory) or BALB/cAnNHsd mice (Envigo) were used in the study in accordance with all federal regulations, NIH guidelines, AAALAC, and IACUC approval.
Wild animals	This study did not include wild animals.
Field-collected samples	This study did not involve samples collected from the field.
Ethics oversight	All animal experiments were performed under the Animal Study Protocols, as reviewed and approved by the Institutional Animal Care and Use Committee of the VRC, NIAID, NIH. All animals were housed and cared for in accordance with local, state, federal, and institutional policies of NIH and American Association for Accreditation of Laboratory Animal Care.

Note that full information on the approval of the study protocol must also be provided in the manuscript.
